# Disseminated Cerebro-Spinal Sclerosis

**Published:** 1885-10

**Authors:** Dyce Duckworth

**Affiliations:** St. Bartholomew’s Hospital


### Disseminated Cerebro-Spinal Sclerosis.

By Dyce Duckworth, M.D., F.R.C.P.
St. Bartholomew's Hospital.

Gentlemen—I bring before you to-day a patient lately admitted to Bed 1, in John ward, whose case furnishes me with some points of great interest, to which I shall ask your attention. It is one of a class well fitted for a clinical lecture, which, as you know, is nothing if not demonstrative, and little more than a systematic or didactic one if the patient is not brought before you. Clinical medicine has all to do with individual cases, and that teaching of it is most proper which best illustrates the points to be noted to each of you in as direct and living a manner as possible.

I will presently tell this man to walk across the theater, and ask you to notice his gait. If you look at him first as he stands at rest, you will not observe anything remarkable about him. You see a young man in seeming good health, well nourished, and with complete control over his equilibrium. I now ask him to close his eyes. He stands erect, and without any tremor or instability. And now, as he walks, you notice a peculiarity in the action of his right leg. This limb moves stiffly, and there is over-action of it. It is lifted higher off the ground than the other, and is clumsy in movement. We say there is some spastic action in it. I now give him this glass of water to take in his right hand. At once, you see, a violent spasmodic action occurs, vigorous tremulation, so great that he nearly empties all the water before he has well seized the glass. If he next attempts to raise the vessel to his lips, the movements become more and more exaggerated, so that all the water is spilt and the empty glass rattles against his teeth. I remove the vessel from his hand, and all the spasm ceases forthwith. As he stands quietly once more, you notice that the right arm remains tranquil and free from tremor. I try his power of grasp in each hand, and find a marked weakness in the right one, although he is a right-handed man. I now lay bare his forearms and compare the condition of his muscles. You observe no signs of wasting; the muscles are well developed and of good and equal tone on both sides. On examining his face, you see that his muscles of expression are stable and free from tremor, his lips firm, and his eye-balls quite steady. His pupils are unequal, certainly, but that is due to the action of atropine in one of them, used to allow examination of the retina of the right eye. No squint; no facial palsy. Testing his sensory functions, we find no abnormal state; all is as it should be. On enquiry as to any subjective sensorial sensation, he assures us all is natural in each of the four extremities. To curtail our further examination, I may add that there is nothing more to be detected by any physical methods we can employ, save that the knee-reflexes are exaggerated, markedly on the right side, while no ankle-clonus can be elicited. We have, then, a seemingly healthy and vigorous young man, whose only troubles are a clumsy, limping gait, due to disorderly action of his right leg, and inability to employ his right hand and arm because of powerful tremulation and disorderly spasm, which come on the instant he directs his will into this extremity; and this is all. Before he leaves the theater, I ask him to repeat a sentence after me. You notice that he speaks clearly and fluently — with a good Wiltshire accent, to be sure, but without any hesitation or difficulty; and yet, again, on protruding his tongue, you find no noteworthy tremor or peculiarity in it. Let us now take up the history of this case; it is very brief:

G. R., aged twenty-one, a groom, was sent up to us by a former pupil of the hospital, and admitted on Feb. 14th. He states that, nine months ago, trembling movements began in his right arm, which prevented him from following his occupation.

Later on, the right leg became affected, so that he could not walk far on account of the weakness in it. Inquiry into his past history revealed no important illness. He had never had rheumatism, and there was no known history of this or of gout in his family. He had never had chorea, although his present ailment was at first believed to be of this nature. There was no neurotic history in his family, no indication of any previous paralytic attack or hemiplegia, no injury of any kind, and no history of fits. His duties entailed exposure to all kinds of weather, but to no extraordinary exposure. Previous to admission, he had been treated, we learned, with arsenic, belladonna, mineral water, bathing at Bath, etc.; all without avail.

You have already noted that the patient appears a healthy and well-nourished man, and that, so long as he makes no voluntary efforts with the limbs of the right side of his body, there is no indication of disease about him. I show you a specimen of his attempt to write his name. After violent efforts to control the right hand, he made this unintelligible series of scrawls. With the left hand, he has learned to write fairly legibly, but slowly and with difficulty, still without any spasm or tremor. He is awkward in setting his right forefinger on any point; thus he makes bad shots at his nose when he tries to touch the end of it, and hardly succeeds in getting near it. Dr. Stevenson has given us a report on the electrical re-actions of the muscles of the affected limbs, and he states that they all re-act normally to both continuous and interrupted currents, and that there is no loss of electro-sensibility. We have seen the exaggerated reflexes at the knees, especially on the affected side; and you may note an increase in the supinator-reflex of the right arm. No fibrillary muscular contractions. On examining the thorax, nothing abnormal is found. The heart-sounds are healthy and sufficiently loud. The urine is natural and the sphincters act perfectly. Special senses not perverted. No vertigo. Knows where his feet and hands are. Retinæ perfectly healthy, and optic discs well-defined. No nystagmus; pupils re-act naturally; no strabismus. No history of syphilis, and no signs of either inherited or acquired disease of this character. No tenderness on percussing the cranium at any point. In trying to follow with his right toes a circle drawn on the floor, he is very clumsy and erratic. He can jump, though with exertion of more force than is necessary for the distance traversed. The difficulty with the right leg is best seen when he tries to run.

We find, on the whole, more negative than positive signs in this man, and yet we have very definite symptoms before us. What is the lesion here, and where is it? What is the diagnosis and what the prognosis, and the best treatment of it? I mentioned that chorea had been at first suspected in the case. Chorea is sometimes one-sided, and often so for a time in many instances. You would not or you should not long be mistaken as to this. You know that choreic movements are incessant except during sleep, and not only elicited by effort, although they are aggravated by voluntary efforts. And you would not expect to meet with a case of one-sided chorea lasting continuously for nine months. We may, therefore, put that aside. You think, perhaps, of another nervous disorder characterized by tremors and paralysis agitans; the shaking palsy (Parkinson's disease) suggests itself to you. Is this the malady before us? Here is Parkinson's own definition, written in 1817; observe if it tallies with our case: "Involuntary tremulous motion with lessened muscular power in parts not in action, and even when supported; with a propensity to bend the trunk forwards and to pass from a walking to a running pace, the senses and intellect being uninjured." This definition will not apply here. The rule is for the tremor to be persistent and constant in shaking palsy, and rather to cease or moderate when action is induced. The contrary is the case here. Action at once induces tremor. The age of this patient is against his being the subject of shaking palsy, this disorder being very rare before forty years of age are reached. Have we here to deal with a case of so-called post-hemiplegic chorea? I think not, because we have no history and no signs of a past attack of hemiplegia, and the characters of this man's tremors, are not those of the disorder I have alluded to. To mention mercurial tremors is sufficient. These are symmetrical, and affect the head, and the signs of mercurialism are always obvious. We can also exclude hysterical tremors and malingering.

We are brought, at last, to consider this case, then, as one of a class known as insular or disseminated cerebro-spinal sclerosis, or Charcot's disease, as it has been called. It is a remarkable example, certainly, because the disorder is, at present —note, I say, *at present, hemiplegic in character, and also manifestly in an early stage.* We do not often see such cases. This is our diagnosis; sclerotic patches situated in the left half of the brain, possibly in the corpus striatum or crus, and possibly in some portion of the medulla spinalis. I should not like to pronounce with greater certainty anything more than this at present, though I might exclude the inferior frontal convolution and parts around the fissure of Sylvius, with some other regions. We may exclude scrofulous and syphilitic disease in the case, and we are in face of the characteristic lesions which are usually found in these cases, and for which I refer you to your studies in morbid histology. The age cf our patient is just that at which this malady declares itself. It is equally common in each sex, and very rare after forty. Exposure to cold has been a commonly assigned cause. In this disease, no muscular *wasting* occurs, although loss of muscular *power* is found, and no electrical changes arise. Paresis precedes the tremors, and the reverse is the case in shaking palsy. The reflexes are exaggerated. I should not omit to point out to you that many symptoms are wanting in this patient to complete the picture of a typical case. Such a one we had fifteen months ago in John ward. For example, one looks for nystagmus, and for certain symptoms referable to disorder in the medulla oblongata, in most of these cases. I never met before with the exact conditions you see in this man; but still I have hardly any hesitation in making my diagnosis.

As to prognosis. This is certainly grave. I surmise that we have so far only early symptoms before us, and that the disease will make sad progress in time. We may fear the onset of paresis and tremors in the sound limbs, and the implication of speech with what are termed bulbar symptoms. The sclerotic process may spread and new patches of it occur in other portions of the cerebro-spinal system, thus setting up new symptoms. The course of the malady is slow, and may occupy from five to ten years. Deceptive periods of improvement may occur from time to time. Too often the disease goes on from bad to worse, till the patient is rendered helpless and bed-ridden, the limbs becoming rigid and paralytic, dementia supervening. Can we do nothing to arrest this terrible process? Must it go on to the bitter end? Alas, the resources of our art are, we must honestly avow, powerless as yet to avert the progress of this terrible malady. Physicians have been very assiduous in elaborating the differential diagnosis of nervous diseases of late years, but, in respect to therapeutics, we have as yet scored few triumphs.

The outlook is bad, and we might almost despair of rendering help. We shall never do this, I hope, but rather strive the harder to find means of arresting this untoward process. No one drug is pre-eminently indicated. I am giving this man mercury, and mean to bring him fully under its influence. He takes three grains of blue pill each night. Not that I am trying to eradicate any syphilitic taint, for, in truth, we know of none in this case. But we know that mercury is a powerful drug, and able to modify nutritional force very materially. We shall do our patient no harm with it. It may be that some of these obscure perversions of growth are evolutional forms of syphilis transmitted from infected ancestry, and so mercury, fully tried, may chance to be of special use. We know, at any rate, that in the peculiar form of systematic sclerosis of the posterior spinal columns, known as the tabes dorsalis—locomotor ataxia—syphilis plays a very prominent part, to the extent, indeed, of eighty per cent., or more, of all cases. Not that the lesion is itself directly syphilitic or gummatous, but that syphilis, as syphilis, seems to predispose to the particular form of sclerotic change in the cord which sets up the disease we know as tabes dorsalis. We are also maintaining the nutrition of this man's nervous system by cod-liver oil and a good diet. Nitrate of silver has been found of use in early stages of this disease. But, for some time to come, I should prefer to use mercury and iodide of potassium, and carefully watch their effects, and I shall bring the results and the further history of this remarkable case before you on a subsequent occasion.—*Lancet.*

---

In the *Journal d'Oculistique et de Chirurgie* for August, is published an account of an attempt recently made by Chiboet to transplant the eye of a rabbit into the human orbit. The patient was a girl, 17 years of age, whose eye was enucleated for staphyloma. The eye of a rabbit was then enucleated and then placed in the orbit of the girl, being retained in place by sutures. The operation was not a success, the cornea being completely destroyed on the fifteenth day. But the result does not discourage the operator, as he attributes his failure to the pressure of the sutures and the delicate structure of the cornea in rabbits. He promises new experiments, in which he hopes not only to make the eye live, but also to restore the power of vision in the transplanted organ.

This operation has been recently performed in this country by Dr. H. W. Bradford, of Boston, August 9, 1885, and was reported in the Boston *Medical and Surgical Journal* for Sept. 15, 1885.

The patient was a seaman, 35 years of age, whose eye had been injured in childhood, and as a consequence there was atrophy of the optic nerve and globe. The rabbit's eye having been placed in the orbit, and retained in situ by sutures, iodoform was dusted over the lids, and a pad of absorbent cotton and a bandage applied. On the seventh day after the operation, the dressings were removed, and on examination a slight haziness of the cornea was observed, the conjunctiva was chemosed, and overlapped the cornea. Some of the sutures were removed and atropine dropped into the eye, when the dressings were re-applied. On the twelfth day, the dressings were again removed, as the patient complained of pain, and the condition of the eye was found to be as follows: "Cornea hazy, but less so than at previous examination; conjunctiva well united around cornea, and to the globe; the exposed sclerotic on outer side had become covered to about half of its extent; each muscle working satisfactorily. At the last reported examination, August 28th, eighteen days after the operation, the condition was as follows: "Conformation and vision good, cornea improving and has cleared peripherally so as to allow the iris to be seen; chemosis of the conjunctiva has disappeared, although the membrane remains congested; exposed sclerotic on outside of eye is practically covered by the conjunctiva; ocular movements in all directions good." The object of this operation was partially experimental, and partly for cosmetic effects, no sight being expected on account of the atrophy of the optic nerve. This operation has been apparently an entire success. As the globe of a fully-developed rabbit's eye will not increase in size, Dr. Bradford suggests that a young dog's eye might be substituted when children are to be operated upon.

